# Sol-Gel Synthesized Amorphous (In_x_Ga_1−x_)_2_O_3_ for UV Photodetection with High Responsivity

**DOI:** 10.3390/s24030787

**Published:** 2024-01-25

**Authors:** Yupeng Zhang, Ruiheng Zhou, Xinyan Liu, Zhengyu Bi, Shengping Ruan, Yan Ma, Xin Li, Caixia Liu, Yu Chen, Jingran Zhou

**Affiliations:** 1 College of Electronic Science & Engineering, Jilin University, Changchun 130012, China; jilinzhangyupeng@126.com (Y.Z.); wangyitianxiashu@163.com (R.Z.); lxy1104371847@163.com (X.L.); 15526550836@163.com (Z.B.); ma_yan@jlu.edu.cn (Y.M.); jude@jlu.edu.cn (X.L.); liucx@jlu.edu.cn (C.L.); zhoujr@jlu.edu.cn (J.Z.); 2 Institute of Semiconductors, Chinese Academy of Sciences, Beijing 100083, China; yuchensemi@163.com

**Keywords:** ultraviolet photodetector, high responsivity, (In_x_Ga_1−x_)_2_O_3_, oxygen vacancies

## Abstract

β-Ga_2_O_3_ photodetectors have the advantages of low dark current and strong radiation resistance in UV detection. However, the limited photocurrent has restricted their applications. Herein, MSM UV photodetectors based on (In_x_Ga_1−x_)_2_O_3_ (x = 0, 0.1, 0.2, 0.3) by a sol-gel method were fabricated and studied. The doping of indium ions in Ga_2_O_3_ leads to lattice distortion and promotes the formation of oxygen vacancies. The oxygen vacancies in (In_x_Ga_1−x_)_2_O_3_ can be modulated by various proportions of indium, and the increased oxygen vacancies contribute to the enhancement of electron concentration. The results show that the amorphous In_0.4_Ga_1.6_O_3_ photodetector exhibited improved performances, including a high light-to-dark current ratio (2.8 × 10^3^) and high responsivity (739.2 A/W). This work provides a promising semiconductor material In_0.4_Ga_1.6_O_3_ for high-performance MSM UV photodetectors.

## 1. Introduction

Ultraviolet (UV) photodetectors have attracted widespread attention due to their potential applications in solar spectrum detection, UV warning, flame detection, ozone monitoring and so on [1,2,3,4]. Metal oxide semiconductors have attracted considerable attention in the field of ultraviolet photodetectors due to their outstanding electronic and photoelectric properties. β-Ga_2_O_3_ is a semiconductor with a direct wide bandgap of 4.9 eV, excellent chemical and thermal stability, and a high breakdown field strength of 8 MV/cm, second only to diamond, making it suitable for the fabrication of solar-blind ultraviolet photodetectors [1]. Xu et al. fabricated a solar-blind ultraviolet photodetector based on MSM structure using β-Ga_2_O_3_ thin film, which exhibits a low dark current of <10 pA at 10 V [5]. The solar-blind ultraviolet photodetector based on β-Ga_2_O_3_, fabricated by Shen et al., demonstrates a rapid response with a response time and decay time of 0.10 s [6]. However, existing UV photodetectors still have limitations in terms of photocurrent and responsivity. Therefore, enhancing the performance of UV photodetectors has become a focal point of current research [7,8,9,10,11].

Doping is an effective method for improving the performance of UV photodetectors [12,13,14,15]. Doping can increase the carrier concentration in materials, thereby enhancing their conductivity and optoelectronic performance [16,17,18,19,20]. In studies, the photocurrent of Ga_2_O_3_ devices can be improved by doping with In_2_O_3_, which increases the device’s responsivity [21,22]. By altering the In content in (In_x_Ga_1−x_)_2_O_3_ (abbreviated as IGO) thin films, the optical bandgap can be adjusted from 2.9 eV to 4.9 eV, making it useful for the fabrication of ultraviolet and visible-blind photodetectors with different cutoff wavelengths [23]. Simultaneously, the content of In plays a crucial role in regulating the phase transition process of thin films. There is a dearth of literature on IGO-based UV photodetectors. Yoshihiro Kokubun et al. used a sol-gel method to tune the indium (In) content in polycrystalline indium gallium oxide (In_x_Ga_1−x_)_2_O_3_ and found that increasing the In content led to an increase in lattice constant and a decrease in bandgap [24]. Metal-semiconductor-metal (MSM) UV photodetectors were fabricated using radio frequency (RF) sputtered (In_0.9_Ga_0.1_)_2_O_3_ thin films, and it was observed that the device switched from photoconductive to Schottky contact mode with increasing oxygen pressure, with a peak responsivity of 0.31 A/W [23]. Kuan-Yu Chen et al. prepared amorphous indium gallium oxide (InGaO) MSM photodetectors using magnetron sputtering and demonstrated a peak responsivity of 3.83 A/W at 5 V bias and 280 nm illumination [25]. Isa Hatipoglu et al. grew polycrystalline monoclinic indium gallium oxide using metal organic chemical vapor deposition (MOCVD) and tuned the peak responsivity of MSM photodetectors by increasing the indium content incorporated into the Ga_2_O_3_ lattice, from 0.79 A/W (pure Ga_2_O_3_) to 319.1 A/W, 66.1 A/W, and 27.7 A/W for (In_0.203_Ga_0.797_)_2_O_3_, (In_0.177_Ga_0.823_)_2_O_3_, and (In_0.106_Ga_0.894_)_2_O_3_, respectively [26]. However, there is no research yet on effectively controlling the film’s crystal phase by increasing the In content to make the film transition from β-Ga_2_O_3_ phase to an amorphous structure and then to In_2_O_3_ phase. By leveraging the dual effects of crystal phase transition and increased In content, the photodetector’s performance can be improved.

In this study, we prepared IGO UV photodetectors based on a metal-semiconductor-metal (MSM) structure using a sol-gel method. By changing the indium content in the film, the crystal phase of the film can be effectively controlled. Taking advantage of the high oxygen vacancy density of amorphous materials, the lifetime of carriers was significantly extended, and the light-to-dark current suppression ratio as well as the responsivity of the device were improved. Among them, devices prepared with In_0.4_Ga_1.6_O_3_ thin film showed a high light-to-dark current ratio (2.8 × 10^3^) and high responsivity (739.2 A/W). Additionally, this research explores the working mechanisms and performance-influencing factors of the detectors, providing a theoretical basis for further enhancing the performance of UV photodetectors.

## 2. Materials and Methods

**Figure 1:**
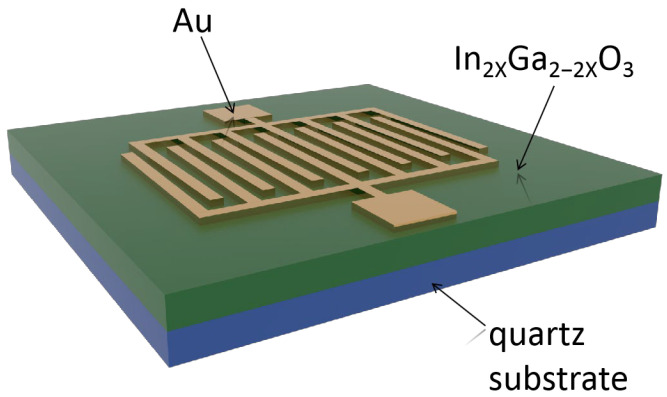
Schematic structure of a metal-semiconductor-metal (MSM) photodetector.

In this work, IGO thin films were prepared on quartz substrate using a sol-gel method. The specific experimental steps are as follows:

A certain amount of gallium nitrate ninahydrate Ga(NO_3_)_3_·xH_2_O (A.R. from Aladdin (Shanghai, China, CAS No. 69365-72-6) and indium nitrate tetrahydrate In(NO_3_)·xH_2_O (A.R. from Aladdin, CAS No. 207398-97-8) were dissolved in 30 mL of deionized water. The metal ions in the solution were controlled at 0.1 M, and the molar ratios of In and Ga were 0:10, 1:9, 2:8 and 3:7, respectively. The corresponding amounts of Ga(NO_3_)_3_·xH_2_O are 3.835 g, 3.452 g, 3.068 g and 2.685 g, and the corresponding amounts of In(NO_3_)_3_·xH_2_O are 0 g, 0.451 g, 0.903 g and 1.654 g. Then, while maintaining a constant temperature of 75 °C, 30 μL of polyoxyethylene lauryl ether (HO(CH_2_CH_2_O)_n_(CH_2_)_11_CH_3_, Brij^®^35, from Aladdin, CAS No. 9002-92-0) was added, and the mixture was stirred for 30 min to obtain the IGO precursor solution. The IGO precursor solution was spin-coated on a pre-cleaned quartz substrate at a rotation speed of 3000 rpm for 30 s, followed by heating at 110 °C for 10 min to complete the gelation process, which was repeated 10 times. After the spin coating was completed, the samples were annealed in a furnace at 700 °C for 30 min. By adjusting the In content in the precursor solution to 0%, 10%, 20%, and 30%, four types of thin films were obtained, namely β-Ga_2_O_3_, In_0.2_Ga_1.8_O_3_, In_0.4_Ga_1.6_O_3_, and In_0.6_Ga_1.4_O_3_. Au interdigital electrodes were fabricated on the thin films using photolithography and radio frequency magnetron sputtering techniques (Vacuum degree was 7 × 10^−3^ Pa, sputter pressure was 2.4 × 10^0^ Pa, sputtering power was 60 W, sputtering time was 6 min, and turntable speed was 10 rpm). From top of view, the finger width and the spacing of the interdigital electrodes were both 20 μm, and the effective detection area of the device was 0.38 mm^2^. The schematic diagram of the IGO MSM photodetector is shown in Figure 1.

The X-ray diffraction patterns of the thin films were observed using Cu Kα radiation on a Shimadzu XRD-6000 diffractometer(Shimadzu, Columbia, MD, USA). X-ray photoelectron spectroscopy (XPS) measurements were carried out using an ESCALAB 250 photoelectron spectrometer (Thermo Fischer, Waltham, MA, USA). Absorption coefficient spectra were measured using a Shimadzu UV-3600 Pharma Spec UV-Vis spectrophotometer. Current-voltage characteristics were measured using a Keithley 2450 source meter (Keithley Instruments, Cleveland, OH, USA). The photodetector’s light response was measured using a monochromator equipped with a 30 W xenon lamp.

## 3. Results and Discussion

**Figure 2:**
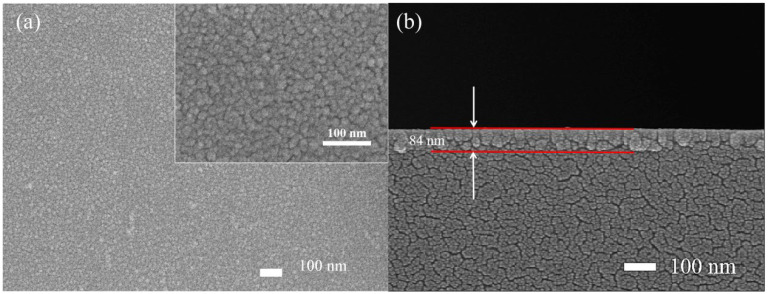
(**a**) Top-view SEM image of the In_0.4_Ga_1.6_O_3_ film, the inset is the high magnification. (**b**) Cross-view SEM image of the In_0.4_Ga_1.6_O_3_ film.

After testing the performance of the prepared UV detectors based on β-Ga_2_O_3_, In_0.2_Ga_1.8_O_3_, In_0.4_Ga_1.6_O_3_ and In_0.6_Ga_1.4_O_3_, it was found that the In_0.4_Ga_1.6_O_3_ detector had the best performance. The SEM images of the In_0.4_Ga_1.6_O_3_ film are presented here, and there is no obvious difference among the SEM images of all films. Figure 2a illustrates the top-view scanning electron microscopy (SEM) images of the In_0.4_Ga_1.6_O_3_ film at different magnification scales. Nanoparticles of In_0.4_Ga_1.6_O_3_, with an average diameter of approximately 15 nm, are densely packed on a quartz substrate, forming a compact film. Figure 2b is the cross-view SEM image of the In_0.4_Ga_1.6_O_3_ film. The thickness is about 84 nm. These results indicate the successful preparation of a flat In_0.4_Ga_1.6_O_3_ film.

**Figure 3:**
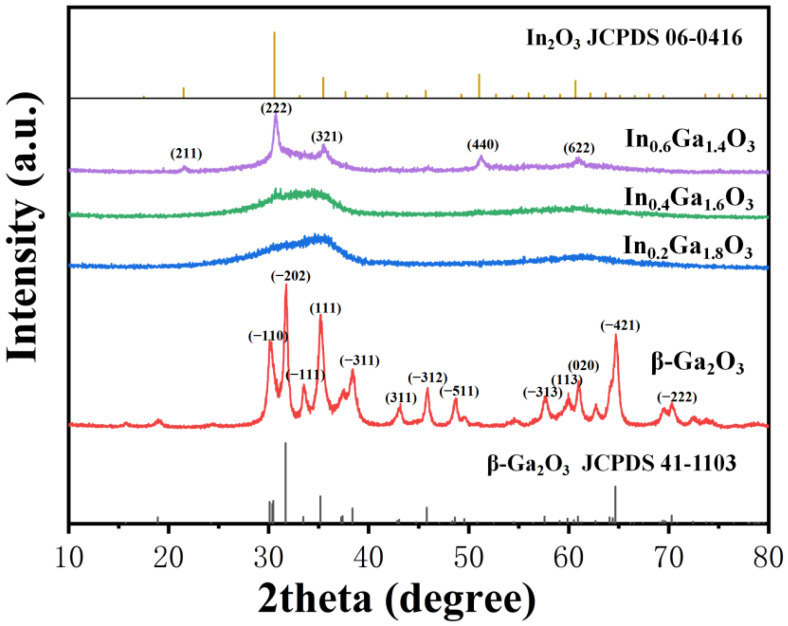
XRD pattern of the β-Ga_2_O_3_,In_0.2_Ga_1.8_O_3_,In_0.4_Ga_1.6_O_3_ and In_0.6_Ga_1.4_O_3_ films.

Figure 3 displays the X-ray diffraction patterns of the IGO thin films. The β-Ga_2_O_3_ film with an In doping concentration of 0% is crystalline, and the observed (−110), (−202), (111), (−311), (−312) and (−421) main peaks are consistent with the β-Ga_2_O_3_ phase, which match well with the JCPDS card number 41-1103 [27]. As the In doping concentration increases, the peak intensities decrease, the full width at half maximum (FWHM) broadens, and the surface crystal quality deteriorates. In the In_0.6_Ga_1.4_O_3_ films, the observed (222), (321), (440), and (622) main peaks are consistent with the In_2_O_3_ phase with the JCPDS card number 06-0416 [28]. As the In doping concentration increases from 0% to 30%, a phase transition occurs from the β-Ga_2_O_3_ phase to the In_2_O_3_ phase [21,29].

**Figure 4:**
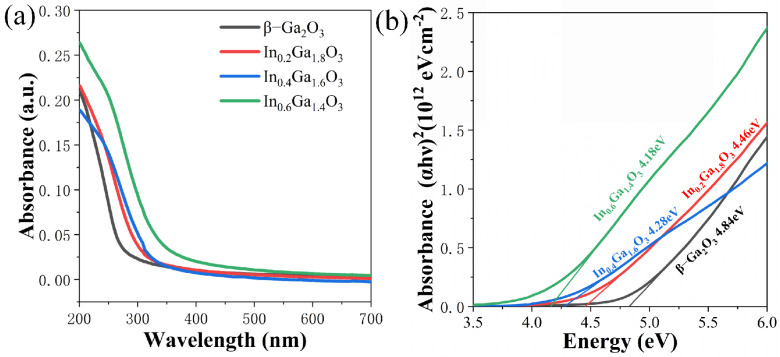
(**a**) Absorbance spectra of β-Ga_2_O_3_, In_0.2_Ga_1.8_O_3_, In_0.4_Ga_1.6_O_3_ and In_0.6_Ga_1.4_O_3_ films. (**b**) Bandgap widths of β-Ga_2_O_3_, In_0.2_Ga_1.8_O_3_, In_0.4_Ga_1.6_O_3_, and In_0.6_Ga_1.4_O_3_ films.

Figure 4a presents the optical absorption spectra of the β-Ga_2_O_3_ and IGO films. All films exhibit significant light absorption in the ultraviolet region. As the In content increases, the absorbance of the films increases, indicating enhanced ultraviolet light absorption by the films. Additionally, it can be roughly observed that as the In content increases, the absorption edge of the curve experiences a redshift. The following formula, Tauc equation, is used to calculate the material’s bandgap width based on the absorption spectra, providing a detailed comparison of how the increase in In content affects the material’s bandgap width [30]:

(1) $$\alpha =A\frac{{\left(h\nu -E_{g}\right)}^{\frac{n}{2}}}{h\nu }$$

where *α* is absorption coefficient, *A* is a constant, *h* is Planck’s constant, *ν* is the frequency of the light, *Eg* is the bandgap energy and the exponent *n* is denoted as the nature of transitions.

As can be seen from Figure 4b, the approximate bandgap width of the thin film materials continuously decreases with increasing In doping concentration. The energy value of the films decreases progressively, with the β-Ga_2_O_3_ film having 4.84 eV, the In_0.2_Ga_1.8_O_3_ film possessing 4.46 eV, the In_0.4_Ga_1.6_O_3_ film exhibiting 4.28 eV, and finally, the In_0.6_Ga_1.4_O_3_ film featuring 4.18 eV, the trend is the same in other researchers’ work [21,26,31].

**Figure 5:**
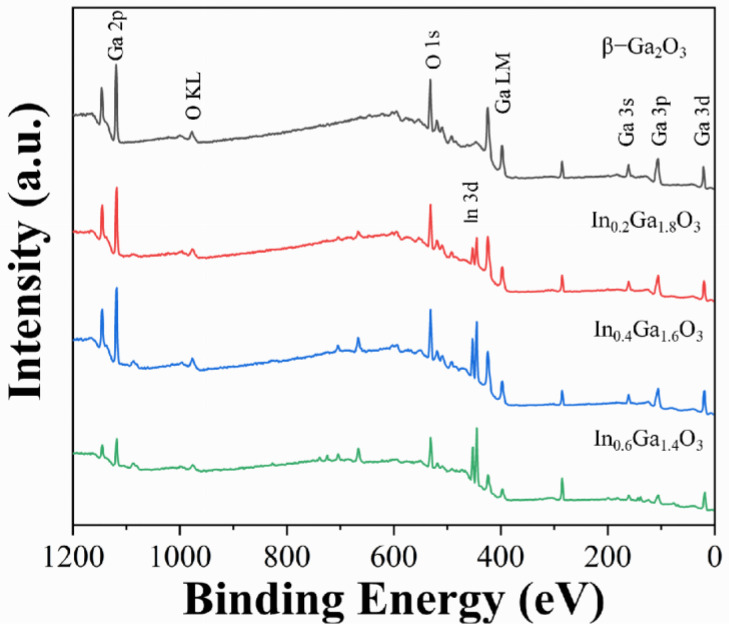
Survey spectra of β-Ga_2_O_3_, In_0.2_Ga_1.8_O_3_, In_0.4_Ga_1.6_O_3_, and In_0.6_Ga_1.4_O_3_ films.

**Figure 6:**
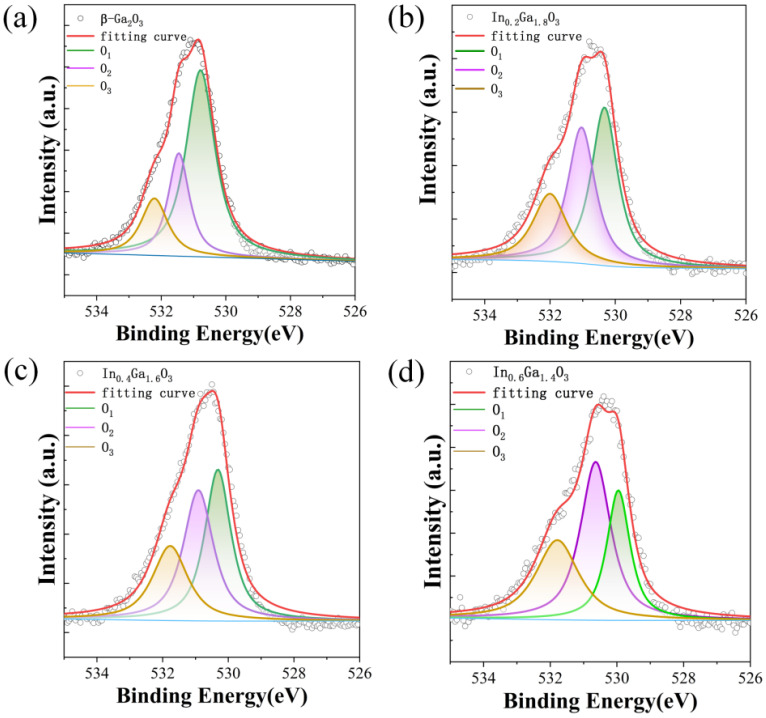
X-ray photoelectron spectroscopy (XPS) spectra of O1s of (**a**) β-Ga_2_O_3_, (**b**) In_0.2_Ga_1.8_O_3_, (**c**) In_0.4_Ga_1.6_O_3_, and (**d**) In_0.6_Ga_1.4_O_3_ films.

The chemical composition of IGO films was investigated using X-ray photoelectron spectroscopy (XPS). Figure 5 shows the full XPS spectrum, and Figure 6 presents the O1s spectra for the four types of thin films. The actual chemical compositions of the β-Ga_2_O_3_, In_0.2_Ga_1.8_O_3_, In_0.4_Ga_1.6_O_3_ and In_0.6_Ga_1.4_O_3_ films are Ga_0.2938_O_0.7062_, In_0.0747_Ga_0.2796_O_0.6457_, In_0.1256_Ga_0.2624_O_0.6124_ and In_0.2092_Ga_0.1761_O_0.6147_ from XPS analysis. With In doping, the amount of In increases from 0.0747, 0.1256 to 0.2093, which is approximately equal to 1:2:3. The amount of Ga decreases from 0.2938, 0.2796, 0.2624 to 0.1761, which is approximately equal to 1:0.9:0.8:0.7. To distinguish the XPS measurements, Gaussian fitting was used to divide the typical O1s peak into three peaks. The deconvoluted peak near 530 eV corresponds to lattice oxygen, while the deconvoluted peak near 531 eV corresponds to defect oxygen [21]. The deconvoluted peak near 532 eV corresponds to adsorbed oxygen [32]. By comparing the four thin film devices, it was found that as the In doping concentration increases, the proportion of lattice oxygen decreases, and the proportion of defect oxygen increases. In the O1s spectrum of In_0.6_Ga_1.4_O_3_, the proportion of lattice oxygen is smaller than that of defect oxygen. Combined with the XRD characterization of In_0.6_Ga_1.4_O_3_, it indicates that as the In content increases to a certain extent, In aggregation occurs on the surface of In_0.6_Ga_1.4_O_3_, altering the chemical environment of the material surface.

**Figure 7:**
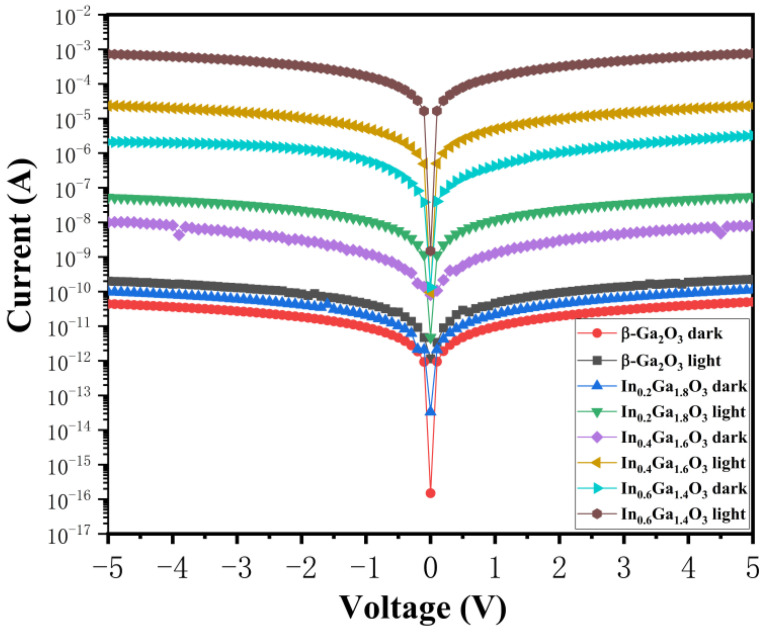
I-V characteristics of β-Ga_2_O_3_ and IGO devices under dark and UV illumination.

Figure 7 displays the I-V curves of β-Ga_2_O_3_ and IGO devices under dark and UV illumination (β-Ga_2_O_3_ @260 nm, 4.8 μW/cm^2^, and IGO @260 and 270 nm, 6.8 and 9.7 μW/cm^2^) at biases ranging from −5 to 5 V. By comparing the photocurrents of the β-Ga_2_O_3_, In_0.2_Ga_1.8_O_3_, In_0.4_Ga_1.6_O_3_, and In_0.6_Ga_1.4_O_3_ devices, it reveals that as the In doping increases, the photocurrent gradually increases, and the increase is quite substantial. At a 5V bias, the photocurrents of the devices with In doping concentrations of 0%, 10%, 20%, and 30% are 0.2 nA, 53.2 nA, 23.4 μA, and 758.5 μA, respectively. It can be observed that as the In doping concentration increases, the rate of increase in the photocurrent of the device slows down. At a 5V bias, the dark currents of the β-Ga_2_O_3_, In_0.2_Ga_1.8_O_3_, In_0.4_Ga_1.6_O_3_, and In_0.6_Ga_1.4_O_3_ devices are 0.05 nA, 0.1 nA, 8.2 nA, and 3.0 μA, respectively. It can be seen that as In doping concentration increases, the rate of increase in the dark current of the device accelerates.

**Figure 8:**
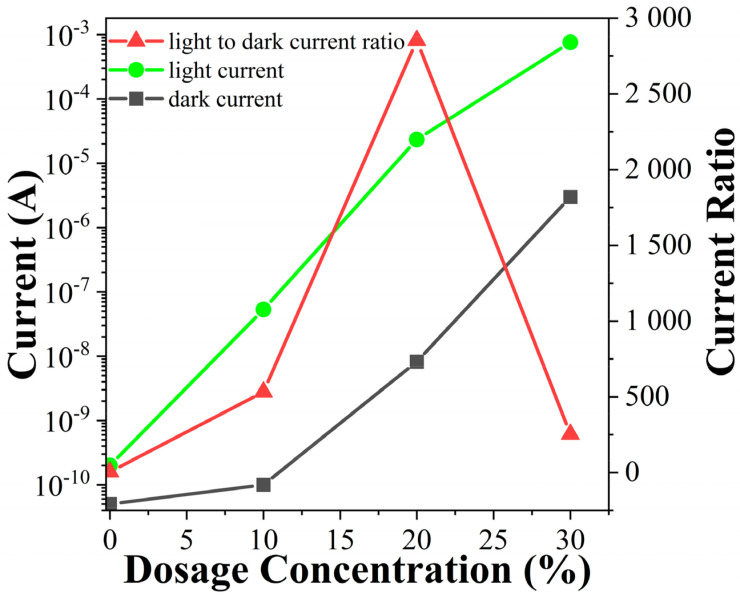
The I_l_, I_d_ and I_l_ to I_d_ ratio values of devices with different In dosage concentration under 5 V bias.

Based on the I-V curves, it was found that as the In doping concentration increases, the photocurrent and dark current of the devices exhibit different rates of increase. To illustrate this trend, Figure 8 shows the light current, dark current, and light-to-dark current ratio of the four devices at a 5V bias. Among them, the In_0.4_Ga_1.6_O_3_ thin film device demonstrates the highest ratio, at 2.8 × 10^3^, which is an order of magnitude higher than the other two devices.

**Figure 9:**
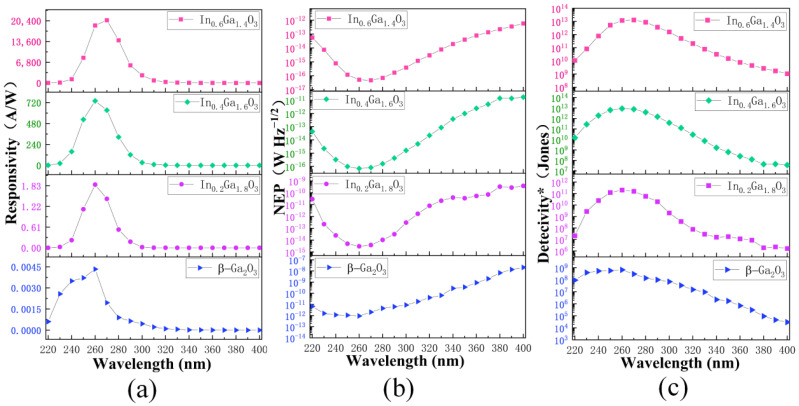
(**a**) Spectral responsivity (**b**) NEP (**c**) Spectral Detectivity* of the β-Ga_2_O_3_ and IGO devices at 5 V bias.

Figure 9a shows the characteristic photoresponse spectra of the photodetector at 5 V bias. The responsivity can be expressed as [33]:

(2) $$R_{\lambda }=(I_{ph}\left(\lambda \right)-I_{d}\left(\lambda \right))/(P\left(\lambda \right)A)$$

where *I_ph_*(*λ*) and *I_d_*(*λ*) are photocurrent and current in dark, *λ* is wavelength of light, *A* and *P*(*λ*) are effective area of the devices and luminous power, respectively. Under 260 nm UV light irradiation, the β-Ga_2_O_3_, In_0.2_Ga_1.8_O_3_ and In_0.4_Ga_1.6_O_3_ devices achieve their maximum responsivity, at 0.00433 A/W, 1.86 A/W and 739.2 A/W, respectively. Under 270 nm UV light irradiation, the In_0.6_Ga_1.4_O_3_ device reaches its maximum responsivity, at 20,579 A/W. Noise equivalent power (*NEP*) is defined as the minimum incident radiation power demanded to realize an SNR (signal to noise ratio) of 1 in a 1 Hz bandwidth. *NEP* can be expressed as [33]:

(3) $$NEP=\sqrt{2qI_{d}}/R(\lambda )$$

The main noise of MSM photodetectors is shot noise, so the mean-square noise current can be shot noise, as in Formula (3). NEP is relevant to a low noise signal and a large responsivity. In Figure 9b the NEP of β-Ga_2_O_3_, In_0.2_Ga_1.8_O_3_ and In_0.4_Ga_1.6_O_3_ devices are 9.23 × 10^−13^ W Hz^−1/2^, 3.04 × 10^−15^ W Hz^−1/2^ and 6.93 × 10^−17^ W Hz^−1/2^ at 260 nm, and the NEP of In_0.6_Ga_1.4_O_3_ device is 4.76 × 10^−17^ W Hz^−1/2^ at 270 nm. The smaller the NEP is, the better the device’s performance. The detectivity* D* represents the ability to detect weak signals from a noise environment, and is calculated by [33]:

(4) $$D=\sqrt{A}/NEP$$

The D* of β-Ga_2_O_3_, In_0.2_Ga_1.8_O_3_ and In_0.4_Ga_1.6_O_3_ devices in Figure 9c are 6.68 × 10^8^ Jones, 2.03 × 10^11^ Jones and 8.89 × 10^12^ Jones under 260 nm UV irradiation. The D* of In_0.6_Ga_1.4_O_3_ device is 1.29 × 10^13^ Jones under 270 nm UV irradiation.

**Figure 10:**
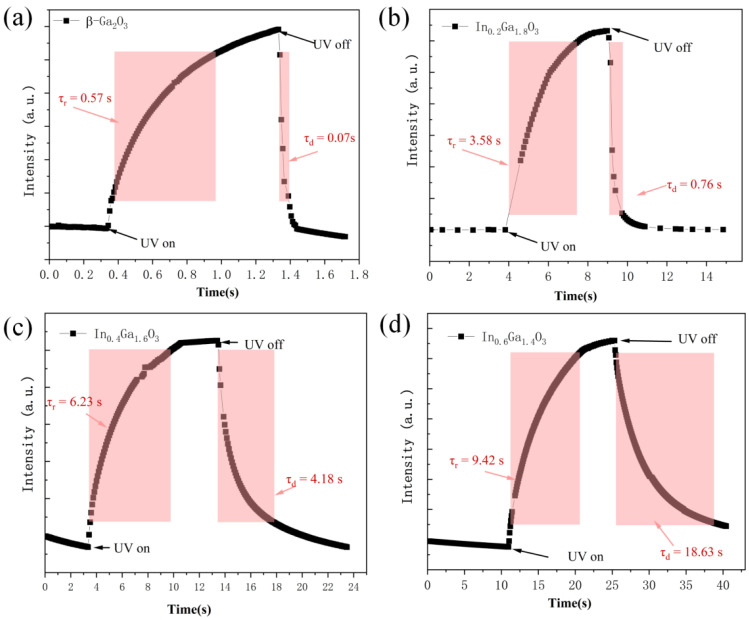
Time response characteristics of (**a**) β-Ga_2_O_3_; (**b**) In_0.2_Ga_1.8_O_3_; (**c**) In_0.4_Ga_1.6_O_3_ and (**d**) In_0.6_Ga_1.4_O_3_ devices.

For IGO photodetectors with a 5V bias applied, Figure 10 shows the rise time (τ_r_) and decay time (τ_d_) of the photocurrent. The response times (from 10% to 90% of the total value) for β-Ga_2_O_3_, In_0.2_Ga_1.8_O_3_, In_0.4_Ga_1.6_O_3_, and In_0.6_Ga_1.4_O_3_ are 0.57 s, 3.58 s, 6.23 s, and 9.42 s, respectively. The recovery times (from 90% to 10% of the total value) are 0.07 s, 0.76 s, 4.18 s, and 18.63 s. The devices exhibit such time response characteristics mainly due to the conduction mechanism of oxygen vacancies. The undoped β-Ga_2_O_3_ thin film device has high crystalline quality and fewer oxygen vacancies, allowing electron-hole pairs to recombine rapidly. In the In-doped thin film devices, the presence of an additional electron layer of the In element compared to the Ga element results in a larger ionic radius, which hinders the crystal growth process of β-Ga_2_O_3_, leading to an amorphous appearance and the generation of more oxygen vacancy defects [34,35]. Therefore, the response and recovery times of the devices increase significantly in thin film devices with different In doping concentrations. The higher the In doping concentration, the more oxygen vacancies are generated, and the longer the response and recovery times of the thin film devices are.

Table 1 is a summary of the parameters of β-Ga_2_O_3_, In_0.2_Ga_1.8_O_3_, In_0.4_Ga_1.6_O_3_, and In_0.6_Ga_1.4_O_3_ MSM UV photodetectors.

**Figure 11:**
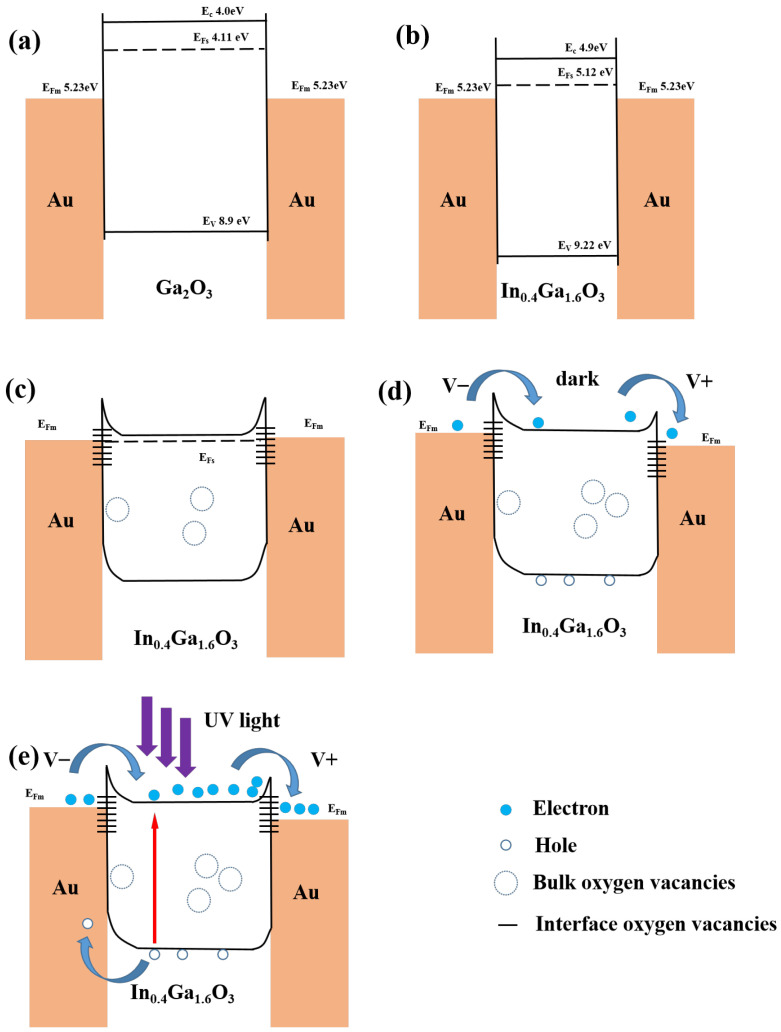
Schematic energy band diagrams of MSM structure. (**a**) Au/Ga_2_O_3_/Au before contact; (**b**) Au/In_0.4_Ga_1.6_O_3_/Au before contact; (**c**) Au/In_0.4_Ga_1.6_O_3_/Au after contact without bias; (**d**) Au/In_0.4_Ga_1.6_O_3_/Au with bias in dark; (**e**) Au/In_0.4_Ga_1.6_O_3_/Au with bias under UV light.

Figure 11 shows the energy band diagram to explain the overall mechanism. Now we discuss the main cases of the In_0.4_Ga_1.6_O_3_ MSM structure. Before contact in Figure 11a,b, the work function of Au is 5.23 eV, and the work functions of β-Ga_2_O_3_ and In_0.4_Ga_1.6_O_3_ are 4.11 eV and 5.12 eV, respectively, which are both higher than those of Au, and thus forming Schottky contacts. The electron affinities of β-Ga_2_O_3_ and In_0.4_Ga_1.6_O_3_ are 4.11 eV and 4.9 eV, and the two bandgaps are 4.9 eV and 4.3 eV. Both β-Ga_2_O_3_ and In_2_O_3_ are n-type semiconductors, and the bandgap of In_2_O_3_ is 3.67 eV. With In doping into β-Ga_2_O_3_, the bandgap becomes small and the Fermi level changes from 4.11 eV to 5.12 eV [36,37]. Furthermore, with increasingly higher In doping concentrations, a larger number of oxygen vacancies capture holes, leading to an augmentation of electron transition density [38]. These interface oxygen vacancies contribute to the interface state, which leads to a decrease in the Schottky barrier. After contact with no bias in Figure 11c, there are bulk oxygen vacancies and interface oxygen vacancies in Au/In_0.4_Ga_1.6_O_3_/Au, and the spikes form when both Fermi levels are unified. When a certain bias is applied on the MSM structure, there is always a reverse Schottky diode. Electrons will cross the barrier under bias, in the way of hot electron emission and tunneling, which forms the dark current, as is shown in Figure 11d. The Fermi level of Au in two reverse Schottky junctions will differ with electrons flowing under bias. Under UV light and a certain bias in Figure 11e, photogenerated electron-hole pairs will be separated by the built-in electrical field of Au and In_0.4_Ga_1.6_O_3_, which contributes to the accumulation of carries and thus improves the photocurrent [39]. As the In doping concentration increases from 10% to 20%, the amorphous state of the film increases. The significant increase in photocurrent is mainly attributed to the increase in the density of electron transitions within the film, while the enhancement of the light-to-dark current ratio is attributed to the pronounced increase in photocurrent [40,41]. With the In doping content rising from 20% to 30%, the film transforms from an amorphous state to the In_2_O_3_ phase. The significant increase in dark current of the device is mainly attributed to the increase in electron mobility (bulk oxygen vacancies by In doping) and the narrowing of the Schottky barrier (interface state by interface oxygen vacancies) due to the formation of the In_2_O_3_ phase [42]. The decrease in the light-to-dark current ratio of the device is attributed to the significant increase in dark current. Since the growth rates of photocurrent and dark current are inconsistent, the device with an In doping concentration of 20% has the highest photocurrent-to-dark current suppression ratio.

Table 2 is the performances compared with other GaO-alloy MSM UV photodetectors, which differ among various element compositions and proportions. The In_0.4_Ga_1.6_O_3_ MSM UV photodetector in this work has a relatively high light current, photo to dark current ratio and responsivity, which indicates the In_0.4_Ga_1.6_O_3_ material having a promising future in UV detection.

## 4. Conclusions

In summary, the effects of indium (In) content on IGO thin-film photodetectors were investigated. As the In doping concentration increased, the thin film transitioned from the β-Ga_2_O_3_ phase to amorphous phase, and then to the In_2_O_3_ phase. A device that exhibits high responsivity (739.2 A/W) and a high photocurrent-to-dark-current suppression ratio (2.8 × 10^3^) was prepared by utilizing the In_0.4_Ga_1.6_O_3_ thin film, which is in the intermediate phase transition process. Furthermore, the influence of Schottky barriers and oxygen vacancies on photocurrent and dark current was explored. Devices with superior performance and greater application potential can be developed by improving traditional single-layer devices through elemental doping.

## Figures and Tables

**Table 1:** A summary of the parameters of β-Ga_2_O_3_, In_0.2_Ga_1.8_O_3_, In_0.4_Ga_1.6_O_3_, and In_0.6_Ga_1.4_O_3_ photodetectors.

	β-Ga_2_O_3_	In_0.2_Ga_1.8_O_3_	In_0.4_Ga_1.6_O_3_	In_0.6_Ga_1.4_O_3_
Eg (eV)	4.84	4.46	4.28	4.18
I_light_ (A)	2.0 × 10^−10^	5.32 × 10^−8^	2.34 × 10^−5^	7.59 × 10^−4^
I_dark_ (A)	5.1 × 10^−11^	1.0 × 10^−10^	8.2 × 10^−9^	3.1 × 10^−6^
I_light_ to I_dark_ ratio	4	532	2853	253
R (AW^−1^)	0.00433@260 nm	1.86@260 nm	739.2@260 nm	20,579@270 nm
NEP (W Hz^−1/2^)	9.23 × 10^−13^@260 nm	3.04 × 10^−15^@260 nm	6.93 × 10^−17^@260 nm	4.76 × 10^−17^@270 nm
D* (Jones)	6.68 × 10^8^@260 nm	2.03 × 10^11^@260 nm	8.89 × 10^12^@260 nm	1.29 × 10^13^@270 nm
τ_r_ (s)	0.57	3.58	6.23	9.42
τ_d_ (s)	0.07	0.76	4.18	18.63

**Table 2:** Performances compared with different MSM ultraviolet photodetectors.

Material	V_bias_ (V)	I_l_ (A)	I_l_/I_d_	Responsivity (A/W)	Ref.
InGaO	5	1.9 × 10^−9^	82.6	6.9 × 10^−5^@270 nm	[43]
(In_x_Ga_1−x_)_2_O_3_	5	3.1 × 10^−5^	1.3 × 10^3^	27.7@255 nm	[25]
(Mg_x_Ga_1−x_)_2_O_3_	5	1.4 × 10^−5^	~10^5^	8.9@254 nm	[44]
Mg_0.18_Zn_0.82_O	13	1.2 × 10^−6^	2	0.2@322 nm	[3]
In_0.9_Ga_0.1_O	10	5.0 × 10^−6^	~10^5^	0.31@300 nm	[23]
Ga_2-x_Sn_x_O_3_	50	8.7 × 10^−7^	1.4 × 10^2^	9.6 × 10^−2^@254 nm	[45]
In_0.4_Ga_1.6_O_3_	5	2.3 × 10^−5^	2.8 × 10^3^	739.2@260 nm	this work

## Data Availability

The data presented in this study are available on request from the corresponding author.
